# Neuron tracing from light microscopy images: automation, deep learning and bench testing

**DOI:** 10.1093/bioinformatics/btac712

**Published:** 2022-10-27

**Authors:** Yufeng Liu, Gaoyu Wang, Giorgio A Ascoli, Jiangning Zhou, Lijuan Liu

**Affiliations:** School of Biological Science and Medical Engineering, Southeast University, Nanjing, China; School of Computer Science and Engineering, Southeast University, Nanjing, China; Center for Neural Informatics, Structures, & Plasticity, Krasnow Institute for Advanced Study, George Mason University, Fairfax, VA, USA; Institute of Brain Science, The First Affiliated Hospital of Anhui Medical University, Hefei, China; School of Biological Science and Medical Engineering, Southeast University, Nanjing, China

## Abstract

**Motivation:**

Large-scale neuronal morphologies are essential to neuronal typing, connectivity characterization and brain modeling. It is widely accepted that automation is critical to the production of neuronal morphology. Despite previous survey papers about neuron tracing from light microscopy data in the last decade, thanks to the rapid development of the field, there is a need to update recent progress in a review focusing on new methods and remarkable applications.

**Results:**

This review outlines neuron tracing in various scenarios with the goal to help the community understand and navigate tools and resources. We describe the status, examples and accessibility of automatic neuron tracing. We survey recent advances of the increasingly popular deep-learning enhanced methods. We highlight the semi-automatic methods for single neuron tracing of mammalian whole brains as well as the resulting datasets, each containing thousands of full neuron morphologies. Finally, we exemplify the commonly used datasets and metrics for neuron tracing bench testing.

## 1 Introduction

Neuronal morphology, specifically the neurite arbors of dendrites and axons stemming from the soma, can be represented as a tree-like structure in a more concise digital form compared to the image of the neuron. The generation of this tree is called neuron tracing, also known as neuron reconstruction, which lays the foundation for systematic and quantitative investigation of the nervous system.

**Fig. 1.**
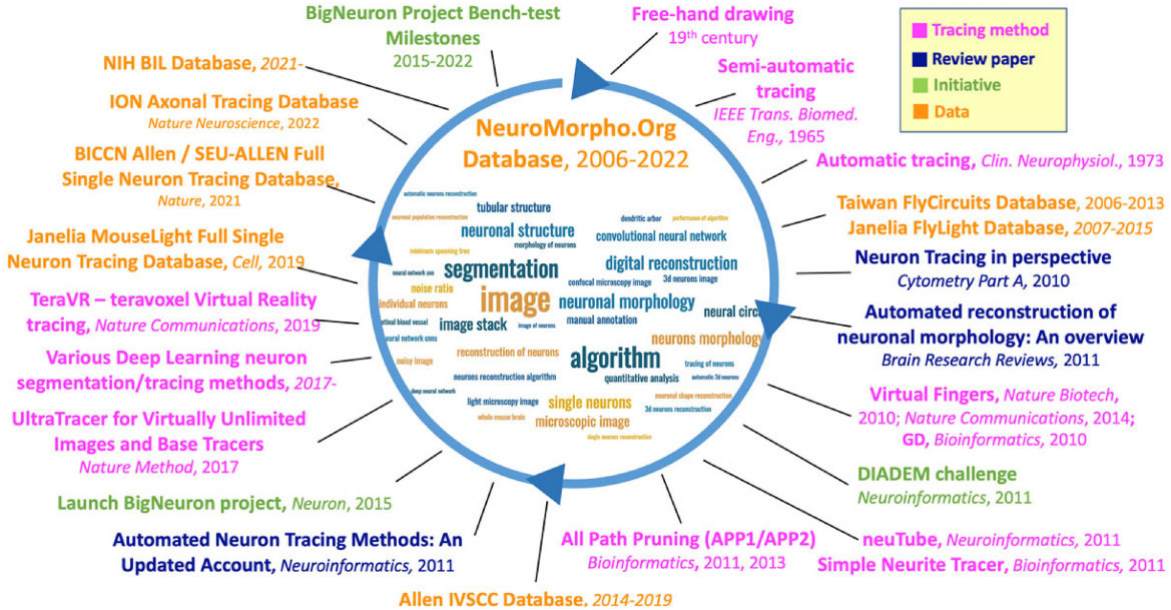
Representative milestones in neuron tracing from light microscopy images. Word cloud in the middle is generated based on the frequency of occurrence in all references in this article. Tracing methods, major previous review articles, recent community initiatives and major datasets/databases are shown in different colors

Figure 1 highlights a small number of selected, highly visible studies of light microscopy-oriented neuron tracing with an emphasis on the last 15 years. At the very beginning, neurites were recorded by time-consuming and labor-intensive free-hand drawings. Semi-automated methods were then introduced by integrating computer-aided algorithms to relieve the vast burden of human labor (Glaser and Van Der Loos, 1965). Fully automatic methods without any manual intervention are in great demand for large-scale data generation and were proposed in the early 1970s (Garvey *et al.*, 1973). Despite the numerous efforts expended since then, there is still a gap between the level of automation and the high-quality tracing required, especially for full morphology tracing of long-projection neurons at the whole-brain level.

The major challenges of automatic methods are the dense arbors of neurites, background noises, fuzzy and inhomogeneous signals along the neurites. Dense arbors may artificially intersect in light microscopic images, leading to crossover structures in the reconstruction. On the other hand, noise and fuzzy signals will lead to early stop of tracing. Many image pre-processing algorithms, noise-insensitive tracing methods and morphology post-processing methods were proposed to alleviate these problems. Powerful feature extraction methods, especially deep-learning-based methods including segmentation and critical point detection, were widely leveraged in recent years.

Full morphology in the mammalian whole-brain containing complete dendritic and axonal arbors is critical for the anatomical and functional characterization of neurons. How to trace the long projection and dense axonal branches introduces additional challenges for whole-brain high-resolution imaging, reconstruction methods and cloud platforms. Until recently, several groups have made breakthroughs in reconstructing full morphologies through the combination of auto-tracing and manual modification. However, the contribution of auto-tracing is in urgent need of improvement.

Several previous survey articles summarized neuron tracing methods (Acciai *et al.*, 2016; Donohue and Ascoli, 2011; Meijering, 2010; Senft, 2011). The last few years have witnessed an explosion of development of new neuron tracing methods (Table 1), especially in two directions, (i) effective discrimination and exclusion of noisy patterns from signals, represented by graph-based pruning methods, such as All-Path Pruning (e.g. Xiao and Peng, 2013) and (ii) sophisticated classifiers that separate noises from signals, represented by recent deep-learning enhanced methods [e.g. Li *et al.* (2017) and Zhou *et al.* (2018)]. In addition, seminal work on scaling-up base tracing methods to virtually unlimited image volume was also developed, such as UltraTracer (Peng *et al.*, 2017). Community collaboration is also becoming a trend, which led to the worldwide BigNeuron project (Peng *et al.*, 2015). Therefore, here, we present an overview of neuron tracing from light microscopy images, focusing on the major milestones in the past few years, including cutting-edge automatic methods, deep-learning-based algorithms, bench testing, databases and single neuron tracing at the mammalian whole-brain level (Fig. 1).

**Table 1.** Accessibility of automatic neuron tracing methods discussed in this review

Methods	Paper	Open source	Platforms
Rodriguez *et al.* (2003)	Rodriguez *et al.* (2003)	Yes	NeuronStudio (Rodriguez *et al.*, 2008)
ORION	Losavio *et al.* (2008)	Yes	ORION
Yuan *et al.* (2009)	Yuan *et al.* (2009)	Yes	FARSIGHT (Luisi *et al.*, 2011)
Neural Circuit Tracer	Chothani *et al.* (2011)	Yes	Neural Circuit Tracer
Open-Curve Snake	Wang *et al.* (2011)	Yes	FARSIGHT
FarSlight Snake	Narayanaswamy *et al.* (2011)	Yes	Vaa3D (Peng *et al.*, 2010b)
RPCT	Bas and Erdogmus (2011)	Yes	Vaa3D
neuTube	Zhao *et al.* (2011)	Yes	Vaa3D & neuTube (Feng *et al.*, 2015)
APP1	Peng *et al.* (2011)	Yes	Vaa3D
Lee *et al.* (2012)	Lee *et al.* (2012)	Yes	Vaa3D & FlyCircuit (Chiang *et al.*, 2011)
APP2	Xiao and Peng (2013)	Yes	Vaa3D
SimpleTracing	Yang *et al.* (2013)	Yes	Vaa3D
Ming *et al.* (2013)	Ming *et al.* (2013)	Yes	flNeuronTool
MOST	Wu *et al.* (2014)	Yes	Vaa3D
Gala *et al.* (2014)	Gala *et al.* (2014)	Yes	Neural Circuit Tracer
SmartTracing	Chen *et al.* (2015)	Yes	Vaa3D
ORION2	Jiménez *et al.* (2015)	Yes	ORION
Neuron Crawler	Zhou *et al.* (2015b)	Yes	Vaa3D
TReMAP	Zhou *et al.* (2016)	Yes	Vaa3D
NeuroGPS-Tree	Quan *et al.* (2016)	Yes	Vaa3D & NeuroGPS-Tree
SparseTracer	Li *et al.* (2017)	No	SparseTracer
ENT	Wang *et al.* (2017)	Yes	Vaa3D
PHD	Radojević and Meijering (2017a)	Yes	ImageJ (Schneider *et al.*, 2012)
UltraTracer	Peng *et al.* (2017)	Yes	Vaa3D
Rivulet2	Liu *et al.* (2018c)	Yes	Vaa3D
FMST	Yang *et al.* (2019)	Yes	Vaa3D
DiMorSC	Wang *et al.* (2018)	Yes	DiMorSC
ShuTu	Jin *et al.* (2019)	Yes	ShuTu
Dai *et al.* (2019)	Dai *et al.* (2019)	Yes	NA
PNR	Radojević and Meijering (2019)	Yes	Vaa3D
CAAT	Huang *et al.* (2021)	Yes	GTree (Zhou *et al.*, 2021)
ViterBrain	Athey *et al.* (2022)	Yes	Brainlit
NeuroStalker	NA	Yes	Vaa3D
LCMBoost	NA	Yes	Vaa3D
Advantra	NA	Yes	Vaa3D
NeuronChaser	NA	Yes	Vaa3D
Axis Analyzer	NA	Yes	Vaa3D
PYZH	NA	Yes	Vaa3D

*Note*: The accessibility of each method is researched based on original paper and respective content on the Internet. This table may not reflect the latest information of a specific method.

## 2 Automatic tracing algorithms

**Fig. 2.**
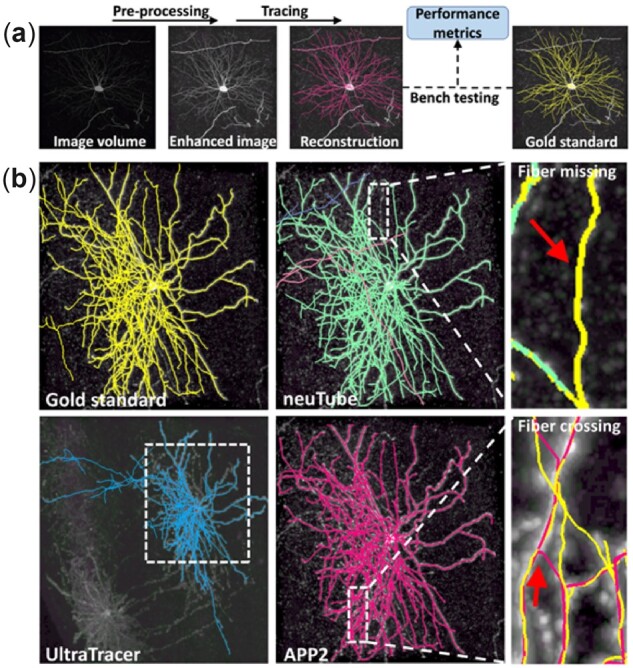
Typical neuron tracing framework. (**a**) Schematic workflow of automatic neuron tracing. The input volume is pre-processed and then traced to obtain a reconstruction. If a gold standard exists, bench testing is applied to evaluate the performance based on metrics. (**b**) Tracing examples of neuTube (local method), APP2 (global method) and UltraTracer (meta method). The boxed area in UltraTracer indicates the same area shown in other examples. Fiber missing and crossing are common errors, which are caused by discontinuous signals and spatially close fibers

A considerable number of automatic algorithms (Acciai *et al.*, 2016; Meijering, 2010) have been proposed since the 1970s and then boosted by initiatives like the DIADEM challenge (Brown *et al.*, 2011) and the BigNeuron project (Peng *et al.*, 2015), which provide standardized datasets, metrics and hackathons. While these algorithms vary greatly in implementation, they share a similar workflow including an optional image pre-processing step and a tracing step that models a tree-like structure from the image (Fig. 2a). The tracing performance is bench tested using many metrics by comparing the reconstructions to ‘gold standards’.

### 2.1 Image pre-processing

Many image pre-processing methods exist for neuronal image processing, with the aim of denoising, illumination correction and fibrous signal enhancement. There are numerous denoising methods, ranging from morphological operations and spatial and frequency domain filters (Buades *et al.*, 2005; Dabov *et al.*, 2006) to more complex methods like sparse coding (Xu *et al.*, 2018), low-rank decomposition (Jin and Ye, 2017) and non-negative matrix factorization-based methods (Guo *et al.*, 2022). Several other methods focus on addressing illumination imbalance in microscopic images, such as CIDRE (Smith *et al.*, 2015), BaSiC (Peng *et al.*, 2017) and AGC (Rahman *et al.*, 2016). For neuronal images, vascular images, or other biomedical images containing vessel-like tissues, methods based on the anisotropic filter (Zhou *et al.*, 2015a) and Hessian Matrix (Frangi *et al.*, 1998; Liang *et al.*, 2017; Mukherjee and Acton, 2015; Sato *et al.*, 1998; Sofka and Stewart, 2006) have been demonstrated to be effective in enhancing tubular structures.

Segmentation as a pre-processing step is becoming more and more popular, such as methods based on the Hessian measurements (Mukherjee *et al.*, 2014; Santamaría-Pang *et al.*, 2015), support vector machine (Chen *et al.*, 2015; Jiménez *et al.*, 2015; Kayasandik *et al.*, 2018), convex optimization (Li *et al.*, 2020) and region-growing (Callara *et al.*, 2020). Besides, deep-learning-based neurite segmentation is demonstrated to be important for high accuracy and robustness, which will be discussed in Section 3.1.

### 2.2 Tracing

Once a neuron image is pre-processed, it will be traced to obtain the tree-like morphology, represented in *swc* (Cannon *et al.*, 1998; Stockley *et al.*, 1993) or *eswc* (Nanda *et al.*, 2018) format. We classify tracing methods into three types, similar to Acciai *et al.* (2016), many of which are summarized in Table 1.

- Local methods where the morphology is reconstructed locally along the extension of signals. As the name indicates, local methods detect putative neurites based on local features, and thus there are prone to get an incorrect topology.
- Global methods detect and connect neuronal nodes or segments based on both local features and global information. The incorporation of global information allows for better discrimination of noises and incorrect connections.
- Meta methods that build on top of existing methods. These methods are orthogonal to base tracers and are often independent modules or frameworks that can combine with any base tracer. In this way, they are always gainful without extra implementation.

#### 2.2.1 Local methods

Local methods usually start from a seed point, either pinpointed manually or detected automatically, and trace greedily along putative fibers estimated based on the signals around the current location. Aylward and Bullitt (2002) defined a ridge criterion based on the Hessian Matrix. Starting from the seed point, the morphology extends to the next rigid point, which is the local maximum in the normal plane shifted from the current point along the approximated tangent direction calculated by Hessian Matrix. Instead of using Eigen analysis of Hessian Matrix, Al-Kofahi *et al.* (2002) leveraged template fitting to determine the tracing direction, where a template contains four parallel edge detectors. Srinivasan *et al.* (2010) employed a moving sphere strategy to gradually fit and propagate through the neurite centerline, the direction of which is computed using the preceding 10 centers, and constrained to a preset angle range to avoid backtracking. The active contour (snake) (Kass *et al.*, 1988) method is proposed by Schmitt *et al.* (2004), where branch points, terminations and cell bodies are manually defined. Wang *et al.* (2011) proposed a tracing framework based on a 3D open-curve snake model, which is an upgraded version of the active contour by initializing branching points automatically with snakes colliding. A recursive principal curve tracing (RPCT) that first detects samples on the 1D principal set of intensity function and iteratively traces the principal curve from the given location is proposed by Bas and Erdogmus (2011). Li *et al.* (2017) proposed a two-stage algorithm SparseTracer using the region-to-region connection method for initial tracing, followed by principal curves estimation to trace the discontinuous neurites. A cylindrical fitting model is introduced in neuTube (Zhao *et al.*, 2011) to sequentially propagate the seed point along the neurite’s principle axis. Ming *et al.* (2013) used a prediction-and-refinement strategy that is based on the exploration of local neuron structural features. MOST (Wu *et al.*, 2014) simulates blood flow and applies a voxel scooping algorithm (Rodriguez *et al.*, 2009) to trace the centerlines from initial seeds. Huang *et al.* (2021) optimized this by using the Content-Aware Adaptive Tracing (CAAT) to trace broken neurites. Rivulet (Zhang *et al.*, 2016) and Rivulet2 (Liu *et al.*, 2018c) iteratively use the fourth-order Runge–Kutta algorithm (*RK4*) for tracking the neuronal arbors from the uncovered furthest potential termini based on the time-crossing map generated by Multi-Stencils Fast Marching. Instead of operating the neuron tracing deterministically, Radojevié *et al.* (2015) and Radojević and Meijering (2017a) proposed methods using Bayesian sequential filtering and Probability Hypothesis Density filtering (PHD) to trace the neuronal structures probabilistically. This approach was further improved by PNR (Radojević and Meijering, 2017b, 2019) and PAT (Skibbe *et al.*, 2019) using Monte Carlo filtering. Zhang *et al.* (2018), Dai *et al.* (2019) and Balaram *et al.* (2019) reformulated the tracing as a behavior problem and introduced a deep reinforcement learning strategy to guide the tracing process. Athey *et al.* (2022) connected the broken components traced by the Bayesian appearance imaging model employing a hidden Markov model. Without awareness of global information, local methods are sensitive to noises and inhomogeneous fibers, which may require integration of the global information (Quan *et al.*, 2016) or an additional post-processing step, such as branch merging (Al-Kofahi *et al.*, 2008) or segment connecting (Liu *et al.*, 2016, 2018c; Zhang *et al.*, 2016).

#### 2.2.2 Global methods

Many global methods extract the skeletons from images and produce a set of unordered skeleton voxels, which are subsequently connected. Cohen *et al.* (1994) proposed a method of sequential segmentation, skeletonization and graph extraction. Critical points including tips, branch points and crossover points are detected from the skeleton, and connected using the volume seed fill operation by this method. To prevent topology collapse in the skeleton, He *et al.* (2003) leveraged an adaptive 3D skeletonization algorithm to prevent erosion of skeletons. Wearne *et al.* (2005) introduced a Rayburst sampling strategy to estimate the branch diameter after image thresholding and skeletonization, also applied tree smoothing and branch points repositioning to optimize the tree. Urban *et al.* (2006) improved the traditional pipeline by combining Otsu binarization and distance transform-based skeletonization.

Yuan *et al.* (2009) employed an intensity-weighted Minimal Spanning Tree algorithm to construct the graph from skeleton points generated by eigenanalysis of the Jacobian Matrix and uses a minimum description length principle to filter out the artifacts introduced in the skeletonization step. Basu *et al.* (2013) and Jin *et al.* (2019) optimized the misconnections by distance and angle-based estimation of interconnections between putative components generated by Hessian-based neurite detection and skeletonization. De *et al.* (2016) formulated the tracing process as label propagation on digraphs, where each node is a filament in the skeleton extracted from the segmentation map, and the directed edge between two nodes represents the corresponding filaments. These skeleton-based methods perform well on high-quality images, while loops and spurs occur frequently when the image quality is poor.

Tracing through seed points detection and connection is another common framework, which often employs Dijkstra’s algorithm to find the shortest path from a starting seed point to other points (Meijering *et al.*, 2003). The method can be optimized using a discrete deformable curve model to achieve more visually appealing tracks (Peng *et al.*, 2010a). The Fast-Marching Method (FMM) (Sethian, 1999), enhanced by weighted distance, is another algorithm employed to find the minimal path by solving the Eikonal equation for a grid map (Benmansour and Cohen, 2011). ORION (Losavio *et al.*, 2008) detects the soma center points and terminations automatically, and then connects them using FMM. Xie *et al.* (2010) and Jiménez *et al.* (2013, 2015) combined seed point detection and shortest-path finding by searching the local intensity maximum and connecting using Dijkstra’s algorithm. Kayasandik *et al.* (2018) optimized the method by integrating prior information, which assumed the neurite orientation changes in a smooth way, and the candidate seeds are searched in a restricted range to alleviate crossover errors. Türetken *et al.* (2012, 2013) optimized the seed point detection according to fibrous structure probability and then find the optimal tree by Mixed Integer Program. Basu and Racoceanu (2014) and Basu *et al.* (2016) employed Gradient Vector Field and FMM to detect critical points and link them based on the speed map. Gala *et al.* (2014) leveraged active learning to reconnect branches dismantled from the tracing generated by FMM from multiple seed points. Wang *et al.* (2018, 2020) extracted the seed points using Discrete Morse Theory, followed by a shortest-path approach to generate a tree. The performance of seed point-based methods depends on the reliability of seed point detection, and the trace may deviate from the centerline of fibers.

Several methods use a graph-based over-tracing and pruning framework, where the neuron is firstly over-traced and then pruned to final morphology. The first version of All-Path Pruning (APP1) was proposed by Peng *et al.* (2011), which builds an over-tracing tree by finding the shortest geodesic paths from the soma location to all foreground voxels using Dijkstra’s algorithm. Redundant nodes are pruned based on the proposed maximal covering minimal-redundant algorithm. APP1 is an orthogonal, substantial derivative of the graph-augmented deformable model (GD), which is a graph-based algorithm that treats every pixel/voxel as a graph vertex and finds the geodesic shortest path between seed points. Different from the bottom-up pruning strategy of APP1, the APP2 algorithm (Xiao and Peng, 2013) accelerates the tracing process through a top-down long-segment-first hierarchical pruning strategy to remove redundant neuronal structures/segments. It also introduced a gray-weighted distance transformation and fast-marching algorithm to improve the robustness and speed. Tang *et al.* (2017) presented an exhaustive neuron tracing framework, in which the neuron is initially traced by over-tracing and redundant branches pruning, followed by an enhanced iteration method to identify the mis-traced structure. FMST (Yang *et al.*, 2019) combines APP1 and MST by recreating the tree generated by APP1 using the MST.

#### 2.2.3 Meta methods

SmartTracing (Chen *et al.*, 2015) introduces a self-learning framework that trains an SVM classifier based on the initial tracing of base tracers, relieving the human intervention of parameter tuning. SmartTracing is a high-level framework that can be applied on top of any base tracers, and can substantially improve their performances. Instead of tracing 3D neuron images directly, TReMAP (Zhou *et al.*, 2016) reconstructs the 2D projections and then reverse-maps the 2D reconstructions into 3D space, using 3D Virtual Finger techniques (Peng *et al.*, 2014b).

Based on the hypothesis that different tracers perform complementarily on different datasets, ENT (Wang *et al.*, 2017) proposed an ensemble framework combining data perturbation and model selection. Base tracers are applied to trace the images differently modified, followed by model selection and ensemble. The best reconstruction is then selected as the output.

Axons may have very long projections to their targeting regions, and sometimes even cross hemispheres. The traversed volumes of these neurons are as huge as billions of voxels for current microscopic images; thus, their full morphology tracing is intractable for most tracing algorithms. To address this issue, Zhou *et al.* (2015b) developed an automatic 3D neuron tracing method called Neuron Crawler, which traces a small image block using APP2 first and propagates to adjacent blocks containing signals connecting to existing fibers. Reconstructed fibers at the boundary regions (10% in width) are discarded to avoid false tracing, and the next block is started from the overlapped region. A subsequent fusion method is designed to avoid over-tracing and topological errors in the overlapping areas. Neuron Crawler has comparable tracing accuracy with much lower memory overhead (<10%) than base tracers. Peng *et al.* (2017) upgraded the framework and proposed UltraTracer. Similar to Neuron Crawler, the initial block is reconstructed by a base tracer, and then the tips close to six boundary faces are detected and pushed into a tip queue. New blocks are estimated and traced based on these tips. This process iterates until no tips are left. In addition, by analyzing the spatial distribution of numerous neuron compartments, prior-based TDAW, which uses adaptive window size for regions of different densities, is introduced for higher efficiency. Inspired by UltraTracer, Wang *et al.* (2018) and Zhao *et al.* (2020) adopted similar block-by-block protocols for large-scale image tracing.

Examples of the three tracing categories are shown in Figure 2b. As a local method, neuTube may be affected by discontinuous signals, which lead to the missing of fibers. APP2 (global method) is more robust in this case but may suffer from fiber crossing for intertwined fibers. The meta method UltraTracer can efficiently trace ultra-volume images at similar accuracy with a low memory and time usage.

Many of these methods are open source and can be accessed through different platforms, among which 3D Visualization-Assisted Analysis (Vaa3D) is the most frequently adopted (Table 1).

## 3 Deep-learning enhanced tracing

Deep-learning methods have shown their superior power in computer vision, natural language processing, recommendation, game playing, etc. Specifically, Convolutional Neural Networks (CNNs) continue to dominate most computer vision tasks and also boost neuron tracing substantially, among which neuronal image segmentation and critical point detection are the two most common applications.

### 3.1 Neuron segmentation

An effective solution to remove noises and bypass inhomogeneous signals is segmentation prior to tracing. Neuron segmentation is conventionally conducted by thresholding, which achieves good performance in high-quality images but is less effective for noisy images. The neural network is more adept in this case. The encoder–decoder architecture of U-Net (Çiçek *et al.*, 2016; Ronneberger *et al.*, 2015) is well suited to this task and is thus gaining popularity. While most of these methods share a similar framework, they differ in the subtle design of architectures, training policy and supervision.

Li *et al.* (2017) is one of the pioneering works utilizing 3D CNN in neuron segmentation by integrating an Inception network (Szegedy *et al.*, 2015) with different kernel sizes and residual structures (He *et al.*, 2016) to learn multiscale representation and alleviate the gradient vanishing problem.

The vanilla 3D CNN model is of great complexity in both memory and time usage, thus several methods are proposed to relieve the requirement of memory and computing capacity. Liu *et al.* (2017) replaced 3D images with 2D projections using a Triple-Crossing 2.5D CNN. Inspired by the development of transfer learning (Hinton *et al.*, 2015; Kong *et al.*, 2018), a knowledge distillation framework is adopted in Wang *et al.* (2019b), in which the large teacher model is used to guide the learning of the small student model to facilitate its training and representation. A method based on the ray-shooting model (Liu *et al.*, 2018a) and dual channel bidirectional LSTM is proposed by Jiang *et al.* (2020), which converts the 3D image-segmentation task into multiple 1D sequence segmentation tasks, where voxel-intensities and boundary-response features of nodes extracted by the ray-shooting model are leveraged to predict the foreground probability of nodes.

Advanced neural network building blocks, such as feature fusion and reasoning modules have demonstrated their power in other fields, and are also adopted for neuron segmentation. The 3D U-Net with multiscale kernels fusion and spatial features fusion is proposed in Wang *et al.* (2019a) to learn different scales of neuronal structure features. Li and Shen (2020) introduced dilated convolutions (Chen *et al.*, 2018) and spatial pyramid pooling layers (He *et al.*, 2015) to capture the global information of the image. Inspired by the great success of multi-head self-attention-based Transformer architectures (Vaswani *et al.*, 2017) for computer vision tasks (Dosovitskiy *et al.*, 2020), Wu *et al.* (2021), Pan *et al.* (2022) and Zhang *et al.* (2022) introduced Transformer into tubular structure segmentation by converting the image features into 1D sequence and modeling both the local contextual information and the long-range dependencies.

The fibrous tree structure of neurons is highly specified, and this domain-specific knowledge is also leveraged in improving segmentation performance. Liu *et al.* (2018b) designed anisotropic convolution kernels to model the anisotropy of image stacks. He *et al.* (2020) optimized the segmentation by removing irrelevant segments and grouping discontinuous segments using a point-cloud network. A network with a graph-based reasoning module (Wang *et al.*, 2021a) and a skeletal loss function clDiceLoss is proposed in Shit *et al.* (2021) to better aggregate information at various levels and model the tree topology globally. A two-stage 3D neuron segmentation approach (Yang *et al.*, 2021a), including a multi-level CNN and a Hessian-repair model, is employed to enhance the weak-signal neuronal structure. To exploit the intrinsic features of voxel points, a voxel-wise cross-volume representation learning method was presented in Wang *et al.* (2021b). SGSNet (Yang *et al.*, 2021b), a two-branch architecture network, unifies neuron-image segmentation and neuronal structure detection into one model to generate continuous segments. A class-aware voxel-wise simple Siamese (Chen and He, 2021) learning paradigm is designed to better learn the latent information for voxels of 3D neuron-image stacks. Li and Shen (2022) proposed a 3D WaveUNet to denoise the 3D neuron image and maintain the structure of nerve fibers. Wang *et al.* (2022) generated the neuronal centerline by learning latent neuron structure distribution using features extracted by the 3D tubular flux model. SRSNet (Zhou *et al.*, 2022), a 3D super-resolution segmentation network, is proposed to acquire high-resolution segmentation images, which enlarges the image by 16-folds to improve the tracing of cross neurites.

The above methods require manual annotated high-quality gold standards, which are difficult to acquire. Several methods have been tried to alleviate the data requirement. Liu *et al.* (2018b) generated synthetic center lines of neuronal structures as labels for subsequent training by applying the Scale-Space Distance Transform to the image. Zhao *et al.* (2019) proposed a progressive framework that combines 3D CNN and traditional neuron tracing algorithms. The pseudo labels are generated by conventional tracing methods and then used to train a CNN model. The procedure is iterated until the converging of the segmentation. Huang *et al.* (2020) produced training labels by automatic tracing methods and then refines them by region-growing and skeletonization methods without manual labeling. Klinghoffer *et al.* (2020) pre-trained the encoder of 3D U-Net by predicting the correct order of permuted slices in a self-supervised way and employed an information-weighted loss function to alleviate the penalization of poor performance on images with few axons. Liu *et al.* (2022) proposed a two-stage image simulation method to generate high-quality image-segmentation pairs for training segmentation networks. In the first stage, prior knowledge is incorporated into a simple model to generate draft image stacks with voxel-wise labels. In the second stage, an MPGAN is applied to adjust the stacks.

### 3.2 Critical point detection

The critical points of neuron structures, including tips, bifurcations and pseudo-crossing points, are topology determinants and are frequently used in graph- or seed-based neuron tracing algorithms.

Many deep-learning-based methods have recently been applied in critical point detection tasks (Chen *et al.*, 2020; Guo *et al.*, 2021; Tan *et al.*, 2019). To improve the efficiency of 3D CNNs-based applications on the 3D volumetric image, Tan *et al.* (2019) proposed a two-level cascaded framework to detect branch points in 3D neuronal images. Candidate regions containing branching points are detected by 3D U-Net. A Multi-View CNN (Su *et al.*, 2015) is used to identify the true branch points from false positives (FPs). Chen *et al.* (2020) applied a 2D multi-stream model to classify the candidates selected on the neuronal skeleton into termination, branching point, crossover point or non-critical point on the basis of features extracted by spherical-patches extraction. Based on these results, a Crossover Structure Separation (CSS) method is presented by Guo *et al.* (2021) to separate the crossover structures. The detected crossover nerve fibers are deformed and separated based on intensity distribution and the angle between crossover fibers in the CSS method.

## 4 Single neuron tracing at whole-brain level

Human brains contain about 86 billion neurons, including large numbers of cross-hemispheric long-projection neurons. The mouse brain is an ideal, tradeoff model for studying human brains. Although neurons are clearly identifiable in sparsely labeled mouse brains, the packaged and intertwined neurites cannot be well reconstructed yet by fully automatic methods in high quality. The majority of mammalian neurons traced were still produced in semi-automatic ways. There are only thousands of high-quality mammalian full reconstructions.

Figure 1 shows a few recent eye-catching studies in this field. The MouseLight project (Winnubst *et al.*, 2019) generated 1000 or so mouse neurons in their full morphology at a submicron scale from two-photon microscopic images, which adopted a semi-automated pipeline to accelerate the reconstruction. The pipeline starts with neurites identification using a pre-trained classifier, and then the derived probability map from the classifier is thresholded, skeletonized and fitted with line segments. To avoid possible crossover structures, all segments are broken at the branching points and crossing points, and connected by annotators. A 3D visualization and annotation platform (Janelia Workstation) (Murphy *et al.*, 2014) is developed to facilitate this procedure by integrating various functionalities including visualization, annotation and proofreading.

**Fig. 3.**
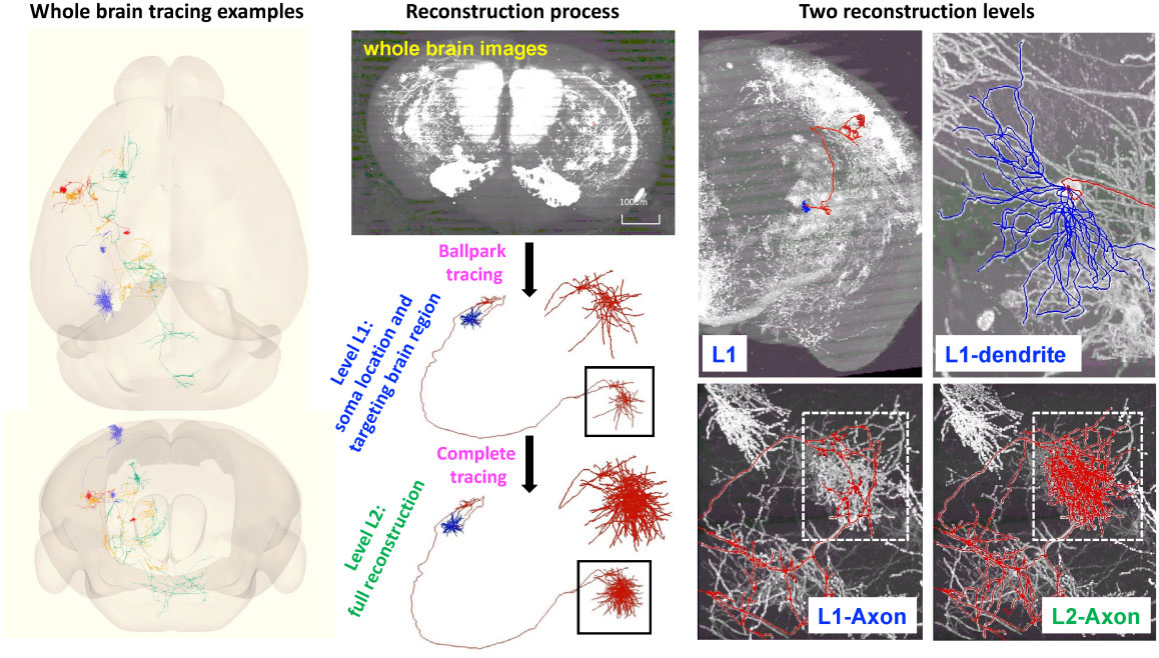
An exemplar neuron tracing/reconstruction application for a mammalian brain’s 3D images. Left panel: examples of reconstruction in whole mouse brain; middle panel: key reconstruction steps, Level 1 (L1) reconstruction provides soma location, dendritic structure and axonal sketch showing targeting brain regions, Level 2 (L2) reconstruction achieves all the traceable neurites based on L1. Right panel: two reconstruction levels with concrete example regions

Peng *et al.* (2021) reconstructed 1741 morphologically diverse single neurons from multiple fluorescence Micro-Optical Section Tomography (fMOST) (Gong *et al.*, 2013)-imaged mouse brains under the BRAIN Initiative Cell Census Network (Ecker *et al.*, 2017) initiative. The reconstructions were accomplished in a semi-automatic way (a key summary of the protocol is shown in Fig. 3), by integrating several intelligent pinpointing algorithms, from points to line segments. The protocol includes two progressive levels of reconstructions: level L1 accomplishes ballpark tracing including the soma location, whole dendritic structure and sketch of the axon, which are mainly produced by a combination of automatic tracing and manual modification. L1 reconstruction answers the neuronal location and targeting regions for biological information. The higher level L2 reconstruction further finishes all the traceable axonal signals. L2 reconstruction supplies the projection strength in every target brain region on the L1 basis. In this study, Virtual Finger (Peng *et al.*, 2014b) was used for fast annotation of fibers by reverse-mapping the annotator’s inputs in the 2D plane of a computer screen to the 3D space. To facilitate neuron tracing on terabyte-scale images, Vaa3D-TeraFly (Bria *et al.*, 2015, 2016) was developed to visualize and manipulate the ultra-large-scale images. TeraVR (Wang *et al.*, 2019c), an open-source virtual reality annotation system, was implemented and made morphology visualization and annotation more precisely from the first-person point of view. All the tools were implemented on the open-source Vaa3D (Peng *et al.*, 2010b, 2014a), which is a cross-platform software for neuroinformatics and brain informatics research.

Gao *et al.* (2022) generated axonal tracing of 6357 neurons in the mouse prefrontal cortex based on fMOST images. A software package, Fast Neurite Tracer (FNT), was developed for neuron tracing and analysis. Large-scale images are firstly split into small cubes similar to Vaa3D-TeraFly blocks. The FNT-tracer package is then used in a semi-automatic style in three steps: finding a putative path by Dijkstra’s algorithm between the start position and target position located by the annotator, similar to GD (Peng *et al.*, 2010b), evaluating the path by comparing it to real fiber signals of neuron structure.

## 5 Bench testing: datasets and metrics

### 5.1 Datasets

The conventional way to evaluate the performance of an automatic algorithm is to compare its reconstructions with corresponding gold standards, which is similar to the ground truth in machine learning. In general, a loosely defined ‘gold standard’ dataset contains expert-annotated reconstructions, which are supposed to be confident to some degree. As shown in Figure 1, a centralized public neuron structure database NeuroMorpho.Org (Ascoli *et al.*, 2007) is to date the largest neuron morphology repository containing 185 949 morphologies contributed from over 900 research labs worldwide. Several other databases also archived a considerable number of high-quality reconstructions, e.g. FlyCircuits (Chiang *et al.*, 2011) and FlyLight (Jenett *et al.*, 2012) contain over 20 000 reconstructions and primary neuronal images in *Drosophila* brain. Researchers at Allen Institute released *In Vitro* Single Cell Characterization database for human and mouse neurons (Wang *et al.*, 2020), which integrates electrophysiological, morphological, histological, transcriptomic data etc. The NIH Brain Image Library database (Benninger *et al.*, 2020) archived over 6000 brain image entries of various organisms and modalities. These databases can be conveniently accessed for sharing, mining and interacting through their web interface. About 400 high-quality neuronal images and their corresponding gold standards are maintained in DIADEM (Brown *et al.*, 2011) and BigNeuron project (Peng *et al.*, 2015), which contains a number of species (e.g. fruitfly, silk moth, dragonfly, zebrafish, Xenopus, chick, mouse, rat and human) and anatomical regions (cortical and subcortical areas, retina and peripheral nervous system). Synthetic data are also a good starting point dataset for prototyping new algorithms due to their correctness and simplicity. These synthetic neuronal images are usually generated according to pre-defined morphologies (Radojević and Meijering, 2019; Vasilkoski and Stepanyants, 2009).

The algorithms can be bench tested according to the similarity of reconstructions to gold standards and calibrated by metrics.

### 5.2 Distance metrics

Distance metrics are widely employed, by calculating the node-wise minimal distances for all nodes in subject morphology to the gold standard. Practically, the two morphologies should be uniformly resampled to guarantee the distances between two connecting nodes are of the same spatial distance (SD). SD, one of the most commonly used metrics, is computed by averaging the reciprocal minimal Euclidean distances of nodes in two morphologies. Substantial spatial distance is defined as the average SD of nodes with SDs greater than some distance threshold, usually two voxels, to remove the positional deviations. The percentile of different structures (Peng *et al.*, 2011) is also a frequently used metric, in which the different structure refers to nodes that have a minimal distance larger than the defined distance threshold. On top of these distance metrics, statistical metrics precision, recall and *F*1-score are also used (Liu *et al.*, 2018c). A node is regarded as a true positive (TP) if at least one node in the gold standard has a distance of fewer than several voxels (e.g. 4), otherwise, it is a FP. The false negative (FN) is defined similarly. The precision is computed as

$$\mathrm{Precision}=\frac{\mathrm{TP}}{\mathrm{TP}+\mathrm{FP}}$$

while the recall is defined as

$$\mathrm{Recall}=\frac{\mathrm{TP}}{\mathrm{TP}+\mathrm{FN}}.$$

The *F*1-score balances precision and recall as

$$F_{1}=2\times \frac{\mathrm{Precision}\times \mathrm{Recall}}{\mathrm{Precision}+\mathrm{Recall}}.$$

Distance metrics evaluate the reconstructions using geometric distance but ignore the connectivity of the morphology, thus insensitive to the topology errors.

### 5.3 Topology metrics

Some topology metrics rely on the matching of topological components, including paths and subgraphs. The DIADEM metric (Gillette *et al.*, 2011) was the default metric in the DIADEM challenge. It is widely used to measure the morphological similarity between two morphologies by matching the locations of bifurcations, terminations and the topology between them. To compute the correspondence of the critical points between the gold standard and the subject reconstruction, the corresponding node in the automatic tracing is searched in a cylindrical region around the node for each node in the gold standard. Path length error is calculated to determine the matches between the gold standard paths and the traced paths, based on geometric deviations between them. Path2Path (Basu *et al.*, 2011) is a path matching method, which decomposes a neuron hierarchically into paths and calculates the minimum geometric deformation from paths in one neuron to the other. The path deformation energy is estimated as the SD of the path between two neurons, which combines hierarchical path level and path concurrence. NetMets (Mayerich *et al.*, 2012) compares both the geometry and connectivity of the two traces using four normalized values based on seed points mapping and path matching: geometric FN rate, geometric FP rate, connective FN rate and connective FP rate.

Instead of measuring the morphological similarity through component matching, some metrics calculate the topological features of each neuron and map them into subspace as a feature vector or matrix. Li *et al.* (2017) proposed a topological persistence-based vectorization framework, which encodes a neuron into a 1D feature vector. Ljungquist *et al.* (2022) optimized the method by combing the morphometrical characteristics calculated by L-Measure (Scorcioni *et al.*, 2008), followed by a maximum likelihood-based automatic dimensionality selection using principal component analysis. Topological Morphology Descriptor (Kanari *et al.*, 2018) maps each branch of the morphology to a lifetime line connecting the start and end points of the branch. The lines are arranged based on some ordering function, resulting in a unique ‘barcode’ signature.

In addition to the metrics mentioned above, metrics for vessel-like structure evaluation can also be adapted to neurons. For instance, Mut *et al.* (2014) employed the distribution of morphological characteristics for morphological similarity estimation. Another three metrics, OPT-P, OPT-J and OPT-G were proposed (Citraro *et al.*, 2020) for road evaluation, which are based on path, junction and subgraph, respectively.

## 6 Conclusion

Large-scale neuron morphologies are critical for delineating the mechanism of brain function, neuronal types and circuit connectivity, which call for reconstruction in a fully automatic way. The dense packing of neurite arbors, noisy and inhomogeneous signals in current light microscopic images make the automatic methods hard to produce accurate tracing. Deep-learning methods can improve accuracy and robustness, but it still has a long way to go. Given the imperfect neuronal images, one practical way might be to incorporate as much domain knowledge of neuron morphology, either from existing reconstructions or biological insights, and tracing progressively and comprehensively like an expert.

Mammals including mice and non-human primates are good model animals for human brain studies because of their functional conservation and much easier feasibility. Several frameworks, e.g. Neuron Crawler and UltraTracer, were proposed to tackle the tracing of long-projection neurons that widely exist in mammalian brains. These frameworks share a similar block-by-block design; however, they could not produce quantitative analyses accurately enough. All complete neurons for mammalian whole brains were generated semi-automatically to date. To foster the development of neuron tracing algorithms, various initiatives including DIADEM and BigNeuron were organized. Standardized metrics and datasets were provided for critical benchmarking and comparing in DIADEM and BigNeuron.

Except for the tracing methods, cloud platforms and tools, which are applicable for ultra-scale images and metadata visualization, collaborative manipulation and interactive analyses are equally important for large-scale morphology generation. These platforms could provide gold standards resources and ground truth for tracing algorithms tracing and quality control of reconstructions. Existing platforms and tools are not well prepared for such ultra-scale neuronal data processing and community collaboration is in demand.

Nevertheless, compared with 10 years ago, we believe the proposed high-throughput neuron reconstruction has greatly evolved and could be achieved in the near future. With the rapid development of imaging and automation, we believe that neuron tracing from light microscopy images can be of much higher quality in the next decade.

### Author contributions

Y.L. and L.L. designed the overall framework, drew the figures and revised the manuscript. G.W. collected most of the materials and drafted the first version. G.A.A. assisted with the overall framework and edited the manuscript. J.Z. and L.L. collaborated in whole-brain imaging collection.

## Funding

This work was supported by Southeast University (SEU) to support informatics data management and analysis pipeline of full neuronal reconstruction platform. This work was also supported by a MOST (China) Brain Research Project, ‘Mammalian Whole Brain Mesoscopic Stereotaxic 3D Atlas’ [2022ZD0205200 and 2022ZD0205204]. G.A.A. acknowledges funding from NIH grants [R01NS36000, RF1MH128693 and R01NS86082].

*Conflict of Interest*: none declared.
